# Usage Patterns of Stop Smoking Medications in Australia, Canada, the United Kingdom, and the United States: Findings from the 2006–2008 International Tobacco Control (ITC) Four Country Survey

**DOI:** 10.3390/ijerph8010222

**Published:** 2011-01-20

**Authors:** Brian V. Fix, Andrew Hyland, Cheryl Rivard, Ann McNeill, Geoffrey T. Fong, Ron Borland, David Hammond, K. Michael Cummings

**Affiliations:** 1 Department of Health Behavior, Roswell Park Cancer Institute, Elm and Carlton Street, Buffalo, NY 14263, USA; E-Mails: andrew.hyland@roswellpark.org (A.H.); cheryl.rivard@roswellpark.org (C.R.); michael.cummings@roswellpark.org (K.M.C.); 2 Division of Epidemiology and Public Health, University of Nottingham, Nottingham, NG5 1PB, UK; E-Mail: ann.mcneill@nottingham.ac.uk; 3 Department of Psychology, University of Waterloo, Waterloo, ON, N2L 3G1, Canada; E-Mail: gfong@watarts.uwaterloo.ca; 4 Ontario Institute for Cancer Research, Toronto, ON, M5G 1L7, Canada; 5 The Cancer Council Victoria, Carlton, VIC, 3053, Australia; E-Mail: ron.borland@cancervic.org.au; 6 Department of Health Studies and Gerontology, University of Waterloo, Waterloo, ON, N2L 3G1, Canada; E-Mail: dhammond@uwaterloo.ca

**Keywords:** tobacco cessation, ITC Survey, varenicline

## Abstract

Varenicline is a new prescription stop smoking medication (SSM) that has been available in the United States since August 1, 2006, in the United Kingdom and other European Union countries since December 5, 2006, in Canada since April 12, 2007, and in Australia since January 1, 2008. There are few population-based studies that have examined use rates of varenicline and other stop smoking medications. We report data from the ITC Four Country survey conducted with smokers in the US, UK, Canada, and Australia who reported an attempt to quit smoking in past year in the 2006 survey (n = 4,022 participants), 2007 (n = 3,790 participants), and 2008 surveys (n = 2,735 participants) Respondents reported use of various stop smoking medications to quit smoking at each survey wave, along with demographic and smoker characteristics. The self-reported use of any stop smoking medication has increased significantly over the 3 year period in all 4 countries, with the sharpest increase occurring in the United States. Varenicline has become the second most used stop smoking medication, behind NRT, in all 4 countries since being introduced. Between 2006 and 2008, varenicline use rates increased from 0.4% to 21.7% in the US, 0.0% to 14.8% in Canada, 0.0% to 14.5% in Australia, and 0.0% to 4.4% in the UK. In contrast, use of NRT and bupropion remained constant in each country. Males and non-whites were significantly less likely to report using any SSM, while more educated smokers were significantly more likely to use any SSM, including varenicline. Our findings suggest that the introduction of varenicline led to an increase in the number of smokers who used evidence-based treatment during their quit attempts, rather than simply gaining market share at the expense of other medications. From a public health perspective, messages regarding increased success rates among medication users and the relative safety of stop smoking medications should be disseminated widely so as to reach all smokers of all socioeconomic classifications equally.

## 1. Introduction

Pharmacological treatments such as nicotine replacement therapy (NRT), sustained-release bupropion, and varenicline are effective in terms of diminishing the severity of withdrawal symptoms and reducing the chance of relapse in smokers who make a quit attempt [1–4]. Despite this evidence, the majority of smokers today still make their quit attempts without using any form of assistance, although the proportion has increased slightly over the past two decades [5]. Approximately 3–5% of smokers who make an unaided quit attempt report remaining smoke free one year following their quit attempt [1]. In the United Kingdom, an evaluation of the National Health Service (NHS) Stop Smoking Service program indicated that smokers who attempted to quit using stop smoking medication and behavioral support provided through the NHS services were nearly 4 times more likely to be quit at 52 weeks than smokers who attempted to quit with no assistance [6].

Given the number of pharmacotherapies shown to be effective in aiding smoking cessation attempts, United States Clinical Practice Guidelines recommend the use of pharmacotherapy for all smokers who want to make a quit attempt, so as long as no contraindications for medication use are present [5,7]. In the United Kingdom, the government provides stop smoking medications through the National Health Service prescription program [6] and the NHS Stop Smoking Services. In Australia, there are no subsidies on the use of NRT, but prescription only medications are subsidized for one course of treatment per year, requiring two prescriptions at a small cost to the individual [8]. In Canada, stop smoking medications may be obtained on prescription or over the counter. Although no national-level guidelines exist, some Canadian provinces do offer comprehensive subsidy programs for smokers who wish to use pharmacotherapy in an attempt to stop smoking [9].

Historically, the most common pharmacotherapy used in smoking cessation was nicotine replacement medication, such as the nicotine patch and nicotine gum [10,11]. NRT in the form of gum, patch, and lozenge has been available over the counter without a prescription in the United States since 1996 [12]. The United Kingdom National Health Service has provided NRT on prescription since April 2001 [6] but NRT products are also available over the counter without a prescription in pharmacies and in other retail outlets. In Australia, the various forms of NRT became available over the counter from pharmacies from 1997 to 2003, with some forms becoming available in ordinary stores from 2005, although NRT manufacturers were slow to use this channel, and it still little used [8]. In Canada, NRT has been available over the counter without a prescription since 1998 [9].

Sustained-release bupropion is an atypical antidepressant approved for use in smoking cessation. In the United States, bupropion has been approved for use in smoking cessation since 1997. The United Kingdom National Health Service has provided bupropion on prescription since June 2000 [6]. It has also been available on prescription in Australia since 2000 [8]. Health Canada approved bupropion for use via prescription in May 1998 [9].

Varenicline, a partial agonist of the nicotinic acetylcholine receptor, (Chantix® in the United States and Champix® in the United Kingdom, Australia, and Canada) is a prescription stop smoking medication; clinical studies suggest that varenicline has demonstrated clinical efficacy as a stop smoking medication with relative risks for quitting approximately 2.5–3 times that of a placebo [13–15]. Varenicline has been available in the United States since August 1, 2006 and in the United Kingdom since December 5, 2006 [16]. In Canada, varenicline was has been available since April 12, 2007, and since January 1, 2008 in Australia.

While the clinical studies demonstrate the efficacy of varenicline, few population-based studies that examine the prevalence of use of this medication, whether it is displacing users of other stop smoking medications, and the characteristics of smokers who report the use of medication as part of a quit attempt.

This study uses data from the International Tobacco Control (ITC) 4 Country Survey to examine trends in use of stop smoking medications between 2006 and 2008 in the United States, United Kingdom, Australia, and Canada, corresponding to the time period when varenicline was made available as a government approved stop smoking medication in each of these four countries. The overall objective of the study is to examine usage patterns of stop smoking medications in all four countries, with a specific aim of examining how the introduction of varenicline into a market influenced usage patterns for other approved stop smoking medications. Usage patterns of stop smoking medications are examined to determine whether overall use has increased over time or whether smokers predisposed to using stop smoking medications have shifted in terms of the type of medications they are using. Additionally, characteristics of users of different types of stop smoking medications are compared.

## 2. Experimental Section

The data source for this study is the International Tobacco Control (ITC) 4 Country Survey. The objective of the International Tobacco Control (ITC) Project is to evaluate effects of national-level and sub-national tobacco control policies [17].

The ITC Project is a prospective cohort study consisting of surveys in multiple countries. Annual surveys of nationally representative samples of smokers have been completed approximately annually in Australia, Canada, United Kingdom, and United States since 2002. The ITC Survey asks participants questions regarding their smoking behavior, attempts at cessation, and attitudes and beliefs about tobacco products, as well as questions pertaining to each of the demand reduction policies of the Framework Convention on Tobacco Control (FCTC). Cohort members who are lost to follow-up are replaced with newly recruited participants to preserve the overall sample size from wave to wave, thus there is a longitudinal component to the ITC survey as well as a representative cross-sectional component. This study relies on the cross-sectional component of the ITC survey to describe the use of stop smoking medications in these four countries.

For the purposes analyzing medication use patterns, each wave of data was treated as an independent cross-section. We report data on current smokers (and recent quitters) who reported a quit attempt in the year prior to the survey (n = 4,022 in the 2006 wave, n = 3,790 in 2007, and n = 2,735 in 2008).

The use of stop smoking medication was calculated based on self-reports. Respondents were asked whether they had used any stop smoking medications to quit smoking within the past 12 months and, if so, they were read a list of stop smoking medications (including nicotine gum, nicotine patch, nicotine lozenge, nicotine tablet, nicotine inhaler, nicotine nasal spray, nicotine water, bupropion, and varenicline) and asked which they had used each one during this time period.

The main outcome assessed in each survey was the use of any stop smoking medication to quit smoking among participants who reported making a quit attempt in the past year. This included current smokers (daily, weekly, or monthly) and recent quitters (quit <1 month, quit 1–6 months, or quit >6 months). Respondents were asked whether they had used any stop smoking medications to quit smoking within the past 12 months.

The characteristics of participants who reported using any stop smoking medications at the 2008 survey were also examined. Variables included in the analysis were sex (male or female), age (18–24, 25–39, 40–54, 55 and older), ethnicity (majority group in each country or non-majority group), Heaviness of Smoking Index (0, 1, 2, 3, 4, 5, 6—based on a combination of time to first cigarette after waking and number of cigarettes smoked per day), income (low, moderate, or high relative to each country), and education level (low, moderate, or high relative to each country).

Data were analyzed using SPSS 15.0 (SPSS Inc., Chicago, IL). Descriptive statistics were used to provide estimates of the prevalence of stop smoking medication use to quit smoking at the time of each survey. A chi-square test was used to assess trends in use of any stop smoking medication, use of NRT, use of bupropion, and use of varenicline between 2006 and 2008. Additionally, three multivariate logistic regression models (controlling for sex, age, ethnicity, heaviness of smoking index (HSI), income, and education, were constructed in order to examine the characteristics of users of any stop smoking medication, NRT, and varenicline users.

## 3. Results

Results from the 2006, 2007, and 2008 ITC 4 country surveys indicate that the self-reported use of any stop smoking medication among participants who reported making a quit attempt has steadily increased over this three year period in all 4 countries (see Table 1). Between 2006 and 2008, any SSM use rates doubled in the United States (24.4% to 48.8%), increased by about 50% in Canada (31.1% to 45.7%) and Australia (31.5% to 45.6%), and increased by 15% in the UK (31.9% to 36.6%).

Results indicate that varenicline has become the second most used stop smoking medication, behind NRT, in all four countries since being introduced onto each of the four markets. In the US, varenicline use in 2008 was comparable to NRT use (21.7% *vs.* 22.2%, respectively). In Canada and Australia, varenicline use went from 0% in 2006 to nearly 15% in 2008. Uptake of varenicline in the UK has been slower with rates increasing from 0% in 2006 to 4.4% in 2008. While varenicline use was increasing in each country between 2006 and 2008, no statistical differences were observed in NRT or bupropion use rates over the same time period in any country.

### 3.1. Characteristics of Medication Users

Three multivariate logistic regression models were constructed in order to examine characteristics of medication users at the time of the 2008 survey. Results examining the characteristics of SSM users are reported on data combined across each country for the 2008 survey. In all of these analyses, the denominator was those reporting a quit attempt in the previous year. Data were combined because models examining interactions of variables included in the model and country did not show any differences. These results are presented in Table 2.

United Kingdom residents were significantly less likely (OR = 0.61; 95%CI: 0.46–0.80) to have reported using any SSM relative to the other three countries. Males (OR = 0.73; 95%CI: 0.60–0.89) and non-whites (OR = 0.55; 95%CI: 0.38–0.81) were also significantly less likely to have used any SSM. More educated smokers were more likely to report using any SSM in the year prior to being surveyed.

Varenicline users were significantly more likely to be United States residents (OR = 1.53; 95%CI: 1.06–2.20) and significantly less likely to be United Kingdom residents (OR = 0.16; 95%CI: 0.09–0.30). Males (OR = 0.66; 95%CI: 0.49–0.90) and minority group members (OR = 0.47; 95%CI: 0.24–0.92) were also significantly less likely to report using varenicline, while more educated participants were significantly more likely to report using varenicline. These results are presented in Table 2.

NRT users were significantly less likely to be males (OR = 0.80; 95%CI: 0.65–0.99). Older smokers (age 25 and up), heavier smokers (as measured by HSI), and more educated smokers were all more likely to report using NRT in a quit attempt.

## 4. Discussion and Conclusions

Following its introduction in all four countries, varenicline has become the second-most widely used stop smoking medication, although uptake has been slower in the United Kingdom relative to the other three countries. Overall stop smoking medication use rates increased among people making quit attempts in each country between 2006 and 2008, with approximately 40% reporting the use of stop smoking medications in 2008. This increase was driven by sharp increases in varenicline while NRT and bupropion use remained relatively stable. Given the clinical trial evidence demonstrating the effectiveness of varenicline, our findings suggest that the introduction of varenicline led to an increase in the number of smokers who used evidence-based treatment during their quit attempts, rather than simply gaining market share at the expense of previously available stop smoking medications.

The reasons for the difference in the slower rate of uptake in the United Kingdom than in the remaining three countries are unclear. It’s possible that UK practitioners were more cautious to prescribe a new smoking cessation drug, or UK smokers more reluctant to request or accept an offer of varenicline, because of adverse publicity that had arisen in the UK tabloid press after bupropion was launched onto the UK market. This publicity caused a reduction in bupropion use which has remained very low ever since [18]. There has also been negative publicity about varenicline (see below). It’s unclear why the UK may be differentially affected, although the negative publicity of bupropion was covered prominently by its second most popular newspaper.

The findings in this study of an overall increase in use of stop smoking medications in the UK contrasts with the findings of other research which examined trends in SSM prescriptions in the UK and found no overall change following the introduction of varenicline, despite an increase in varenicline prescribing [19]. It’s unclear why the two studies have contrasting findings. One possibility is that the studies used different methodologies: in this study self report of SSM usage among quit attempters is used, wheras in the other UK study [19], medical records of prescriptions issued to all patients are considered. Different sources of stop smoking medications are included in the two studies: the ITC data include stop smoking medications purchased over the counter (OTC) in addition to prescriptions. The inclusion of OTC medications in our study may have accounted for the increase in SSMs overall in the UK and this increase may partly be due to the introduction of smokefree legislation in the UK in 2007, as such legislation has been shown to increase SSM purchases over the counter in other countries [20,21].

Recent data [22] examining medication use among Australian smokers found similar trends for medication use over the same three year time period, but found higher proportions of smokers who reported using any stop smoking medication and NRT. Differences in these findings and those reported in this manuscript could be attributable to differences in sampling frame and analytic approach.

Given the research evidence that varenicline is superior to other cessation aids [13–15], some might be disappointed that its use has not been greater. It is possible that reports of adverse events among varenicline users could be limiting the supply of this drug by some prescribers. A previous report indicated that adverse events, which can lead to discontinuation, are commonly reported among varenicline users [23]. However, other data does not suggest that serious adverse events with varenicline have been experienced beyond what would be expected in a large population of users [24].

A recently published study found no association between varenicline use and an increased risk of depression, self-harm, and suicidal thoughts [25]. In 2008, the United States Food and Drug Administration issued a public health advisory and subsequently updated varenicline product labeling after reports surfaced of individuals experiencing neuropsychiatric symptoms including behavioral changes, depressed mood, and suicidal behavior. Similar warnings were released by the European Medicines Agency in December 2007 [26]. It is unclear what impact the resulting media attention has had on varenicline use rates in the US or in other countries. However, despite these warnings and the media attention given to reports of serious adverse events, use of varenicline increased rapidly in the 2–3 years after its introduction.

For the most part, all the differences in medication use were largely attributable to differences in varenicline use. This suggests that use of varenicline might be subject to different factors than other medications. The finding that better educated smokers were more likely to use varenicline might be because they were more likely to ask for it as they are aware of its existence and its effectiveness. From a public health perspective, messages regarding increased success rates among medication users and the relative safety of stop smoking medications should be disseminated widely so as to reach all smokers equally.

One limitation of this study is the relatively small sample size of the number of users of any given medication, which limited statistical power for some analyses, and means our statements of no effect, should be read as no large effects. We do not attempt to explore possible differences in rates of cessation by medication use because of the complexities of controlling for selection effects would have overly complicated this manuscript. Cessation rates for different SSM users will be reported as future waves of ITC data are collected.

Based on the currently available data, we find that varenicline use increased quickly in the US, Canada, and Australia with slower increases in the UK. Trends in the use of NRT and bupropion were relatively stable in all four countries during the period of time between 2006 and 2008. This suggests that the bulk of varenicline use comes from those who would not otherwise be using medications. We do not know whether this is because this group have used other medications previously and failed with them or they are a new group who had been reluctant to use what had been available. It is important to continue to monitor trends in the use of stop smoking medications, including varenicline.

## Figures and Tables

**Table 1:** Use of Stop Smoking Medications 2006, 2007, and 2008.

	
	2006		2007		2008	

	N	%	N	%	N	%
**United States**	**1,025**		**1,021**		**658**	
Any SSM		24.4		33.0		48.8 1
NRT		19.5		16.5		22.2
Bupropion		4.8		3.4		4.3
Varenicline		0.4		13.7		21.7 1
**United Kingdom**	**903**		**843**		**704**	
Any SSM		31.9		36.3		36.6 1
NRT		29.8		32.7		29.1
Bupropion		1.9		1.1		2.0
Varenicline		0.0		2.0		4.4^1^
**Canada**	**975**		**927**		**676**	
Any SSM		31.1		32.3		45.7 1
NRT		26.8		24.1		24.6
Bupropion		4.8		5.5		5.5
Varenicline		0.0		2.3		14.8 1
**Australia**	**1,119**		**999**		**697**	
Any SSM		31.5		35.3		45.6 1
NRT		26.0		29.8		24.5
Bupropion		5.2		5.1		5.6
Varenicline		0.0		0.1^2^		14.5 1

1 indicates statistically significant chi-square for trend at the p < 0.001 level

2 Of 141 respondents interviewed after on market date, 1 participant (0.7%) reported using varenicline

**Table 2:** Characteristics of stop smoking medication users among quitters from the 2008 ITC 4 Country survey.

	Users of Any Stop Smoking Medication					Users of Nicotine Replacement Therapy					Users of Varenicline				
	
	N	% Used Any SSM	OR	Lower CI	Upper CI	N	% Used NRT	OR	Lower CI	Upper CI	N	% Used Varenicline	OR	Lower CI	Upper CI
	
**Country**															
Canada	676	45.7	1.00	ref.		676	24.6	1.00	ref.		676	14.8	1.00	ref.	
United States	658	48.8	1.09	0.83	1.44	658	22.2	0.84	0.61	1.15	658	21.7	**1.53**	**1.06**	**2.20**
United Kingdom	704	36.6	**0.61**	**0.46**	**0.80**	704	29.1	1.18	0.84	1.58	704	4.4	**0.16**	**0.09**	**0.30**
Australia	697	45.6	0.94	0.72	1.23	697	24.5	0.78	0.72	1.32	697	14.5	0.79	0.53	1.17
**Sex**															
Female	1529	46.6	1.00	ref.		1529	27.1	1.00	ref.		1529	14.7	1.00	ref.	
Male	1206	40.9	**0.73**	**0.60**	**0.89**	1206	22.6	**0.80**	**0.65**	**0.99**	1206	12.4	**0.66**	**0.49**	**0.90**
**Age**															
18–24	140	31.4	1.00	ref.		140	22.1	1.00	ref.		140	4.3	1.00	ref.	
25–39	606	44.4	1.52	0.91	2.52	606	28.2	1.56	0.88	2.76	606	10.9	1.21	0.45	3.27
40–54	1101	45.1	1.32	0.80	2.15	1101	24.5	1.16	0.66	2.03	1101	15.5	1.67	0.64	4.35
55 +	888	44.6	1.39	0.84	2.30	888	24.3	1.14	0.64	2.01	888	14.9	1.80	0.68	4.76
**Ethnicity**															
White, English Speaking	2469	45.1	1.00	ref.		2469	25.6	1.00	ref.		2469	14.3	1.00	ref.	
Non-White, Non-English Speaking	248	33.5	**0.55**	**0.38**	**0.81**	248	20.2	0.79	0.52	1.21	248	8.1	**0.47**	**0.24**	**0.92**
**Heaviness of Smoking Index (HSI)**															
0	256	35.9	1.00	ref.		256	22.3	1.00	ref.		256	10.9	1.00	ref.	
1	268	38.4	1.19	0.80	1.77	268	23.5	1.14	0.73	1.80	268	10.1	0.95	0.50	1.80
2	414	46.4	**1.92**	**1.34**	**2.77**	414	30.4	**1.78**	**1.18**	**2.69**	414	12.6	1.35	0.76	2.40
3	616	43.3	**1.64**	**1.17**	**2.31**	616	23.9	1.36	0.92	2.01	616	13.3	1.33	0.78	2.29
4	375	44.3	**1.56**	**1.08**	**2.26**	375	24.3	1.27	0.83	1.95	375	13.1	1.36	0.76	2.44
5	150	48.0	**1.84**	**1.16**	**2.92**	150	29.3	**1.69**	**1.01**	**2.82**	150	14.0	1.25	0.62	2.55
6	56	42.9	1.51	0.79	2.89	56	28.6	1.59	0.78	3.25	56	7.1	**0.55**	**1.15**	**1.98**
**Income**															
Low	718	46.2	1.00	ref.		718	27.3	1.00	ref.		718	12.1	1.00	ref.	
Moderate	839	42.9	0.83	0.66	1.06	839	24.0	0.82	0.63	1.07	839	13.9	1.18	0.81	1.72
High	812	44.5	0.96	0.74	1.23	812	25.0	0.78	0.59	1.03	812	14.2	1.46	0.98	2.17
**Education**															
Low	1340	41.9	1.00	ref.		1340	24.3	1.00	ref.		1340	11.6	1.00	ref.	
Moderate	891	46.4	**1.31**	**1.06**	**1.63**	891	25.0	1.10	0.86	1.40	891	15.6	**1.56**	**1.11**	**2.18**
High	500	45.6	**1.32**	**1.01**	**1.73**	500	27.4	1.26	0.93	1.70	500	16.0	**1.60**	**1.07**	**2.40**

Note: Bold values indicate statistically significant odds ratios
